# Lung Toxicity of Condensed Aerosol from E-CIG Liquids: Influence of the Flavor and the In Vitro Model Used

**DOI:** 10.3390/ijerph14101254

**Published:** 2017-10-20

**Authors:** Rossella Bengalli, Emanuele Ferri, Massimo Labra, Paride Mantecca

**Affiliations:** 1 Department of Earth and Environmental Sciences, Research Center POLARIS (Particulate Matter and Health Risk), 1 Piazza della Scienza, University of Milano Bicocca, 20126 Milan, Italy; rossella.bengalli@unimib.it; 2 TRUSTICERT SRL, via Mazzini 18/C, 22036 Erba, Italy; emanuele.ferri@trusticert.com; 3 Department of Biotechnology and Biosciences, 2 Piazza della Scienza, University of Milano Bicocca, 20126 Milan, Italy; massimo.labra@unimib.it

**Keywords:** e-cigarette, condensed aerosol, e-liquid, inhalation toxicology, in vitro systems

## Abstract

The diffusion of e-cigarette (e-CIG) opens a great scientific and regulatory debate about its safety. The huge number of commercialized devices, e-liquids with almost infinite chemical formulations and the growing market demand for a rapid and efficient toxicity screen system that is able to test all of these references and related aerosols. A consensus on the best protocols for the e-CIG safety assessment is still far to be achieved, since the huge number of variables characterizing these products (e.g., flavoring type and concentration, nicotine concentration, type of the device, including the battery and the atomizer). This suggests that more experimental evidences are needed to support the regulatory frameworks. The present study aims to contribute in this field by testing the effects of condensed aerosols (CAs) from three main e-liquid categories (tobacco, mint, and cinnamon as food-related flavor), with (18 mg/mL) or without nicotine. Two in vitro models, represented by a monoculture of human epithelial alveolar cells and a three-dimensional (3D) co-culture of alveolar and lung microvascular endothelial cells were used. Cell viability, pro-inflammatory cytokines release and alveolar-blood barrier (ABB) integrity were investigated as inhalation toxicity endpoints. Results showed that nicotine itself had almost no influence on the modulation of the toxicity response, while flavor composition did have. The cell viability was significantly decreased in monoculture and ABB after exposure to the mints and cinnamon CAs. The barrier integrity was significantly affected in the ABB after exposure to cytotoxic CAs. With the exception of the significant IL-8 release in the monoculture after Cinnamon exposure, no increase of inflammatory cytokines (IL-8 and MCP-1) release was observed. These findings point out that multiple assays with different in vitro models are able to discriminate the acute inhalation toxicity of CAs from liquids with different flavors, providing the companies and regulatory bodies with useful tools for the preliminary screening of marketable products.

## 1. Introduction

E-cigarettes (e-CIGs) are nowadays widely used as alternative to conventional cigarettes since they are considered less harmful on health [1]. However, the legislation about their manufacturing, promotion, and distribution has been entering recently into force in United States (U.S.) and Europe, and their long-term effects on human health are far to be clarified. The absence of combustion in e-CIGs is obviously the first crucial distinction from conventional cigarettes regarding health implications. Since e-CIGs do not burn tobacco, it is indeed assumed that vaping of e-liquid should be less toxic than the exposure to cigarette smoke. In addition, cigarette’s tobacco is often contaminated by many tumorigenic pollutants (e.g., cadmium, lead, radioactive polonium, and mycotoxins) and it contains toxic compounds (e.g., tar, nitric oxide, alkaloids and nitrosamine), which could have worst effects when smoked. The reduction in consumption of cigarette’s tobacco might ultimately help in limiting the exposure to carcinogenic molecules and particles [2,3].

Concerning e-CIGs, health risks could be based on a high number of variables, especially related to the device system and liquids’ chemical composition. In this context, recent findings pointed out that some e-CIGs vapors contain significant amounts of heavy metals (i.e., nickel and chromium) due to metals release from coils [4,5], as well as aldehydes from the thermal degradation of glycerol [6,7]. Furthermore, it has been demonstrated that different e-CIG flavors could have different effects on health [8,9]. However, to date, there are no shared and universal toxicology testing models to clarify this issue. Only an exhaustive evaluation of those e-CIG characteristics possibly contributing to human toxicity is needed to assess the real health risk associated to the use of this device.

Generally, e-CIGs are composed of three parts, each one potentially affecting consumer’s safety: a battery, a liquid cartridge (filled or refillable with e-liquids), and a vaporizer consisting of a vaporization chamber with a heating unit known as atomizer. Each of these elements could have direct or indirect effect on human health. The power to the coil is supplied by a Li-ion battery. Different voltages supplied by the battery unit largely influence vaporization temperatures, and therefore, the production of hazardous compounds such as acetaldehyde, formaldehyde, and acrolein, ultimately causing toxicity [10].

The liquid cartridge is filled with several liquids with unknown toxicity. The liquid consists in humectants (i.e., glycerin and propylene glycol), synthetic or natural flavor and nicotine at variable concentrations (0–20 mg/mL in EU countries, and 0→50 mg/mL in U.S.) [11,12]. Different brands adopt different strategies for nicotine content. For MarkTen, the level of nicotine varies by product. VUSE e-cigarettes contain 4.8% nicotine (by weight), which corresponds to more than 50 mg/mL. VUSE tanks, set to come out in February 2016, will have different levels of nicotine [13].

Finally, the atomizer, may drastically vary in its construction material, operational modality, and properties of the generated vapors. All of these variables affect the amount, temperature, number and dimension of final particles, and the chemical constituents of the final aerosol.

Among the three elements, the liquid represents the most variable component due to the presence of several hundred flavors with potential toxic effect. Many investigations about the refill fluids used in the cartridge were performed [8]. However, these analyses did not mimic the real situation in which vapors, derived after heating, could have different toxicity effects with respect to the liquid form. Only recent studies have focused on the effects of e-CIG vapors obtained by different vaporizing protocols. Furthermore, the vapor extracts have been tested on a dis-homogeneous group of cell lines, such as murine neuronal stem cells, human embryonic stem cells, human pulmonary fibroblasts, and human vascular endothelial cells [1,8,14,15,16]. These variable testing approaches, performed even on non-target cells and under different experimental conditions, do not permit a reliable comparison of results. Thus, the more suitable assays for screening e-CIG toxicity is currently under debate. Cervellati and co-workers [2] tested e-CIG vapors on A549 lung cell monoculture, while Scheffler and colleagues [17] proposed the use of human bronchial cells as a more reliable in vitro model for the cytotoxic screening. Although both approaches are useful, a more standard in vitro assay is demanded to obtain comparable results and match the regulatory requirements [18]. Recently, scientists have developed new three-dimensional (3D) co-culture models that are representative of the human alveolar-blood barrier (ABB) to be checked and possibly validated in the future for in vitro inhalation toxicology purposes [19,20]. The idea behind the setup of more complex in vitro models is that the toxicity in the lung cannot be simply represented by the response of epithelial cells, but involves a complex interplay among different cell types, including epithelial, endothelial, dendritic, and macrophage ones. The activation of the lung vascular endothelium, possibly culminating in cardiovascular diseases, has been regarded as the major pathway activated by airborne pollutants [21,22,23].

e-CIG-generated mainstream aerosol is characterized by a particle number distribution modes in the 120–165 nm range, which resulted similar to the conventional cigarette [24]. In addition, the dose of particles estimated to reach the tracheobronchial and especially the alveolar region of the lung was estimated to be even double for the e-CIG [25], making the use of in vitro models of the respiratory barrier mandatory.

To better define an effective and standard analytical method to assess the health risk of vaporized e-CIG liquids, we tested the biological responses to condensed aerosols (CAs) on in vitro models of the human alveolar region. Specifically, a monoculture of lung alveolar epithelial cells (A549) and a 3D in vitro reconstructed ABB composed by alveolar cells (NCI-H441) and lung microvascular endothelial cells (HPMEC) were used. The obtained data were compared and discussed when considering the variability of the responses induced by the different flavors, the presence of the nicotine, and the complexity of the tested biological system.

## 2. Material and Methods

### 2.1. Source of Refill Fluids

A total of six ready-to-use e-liquids were purchased from the market (Table 1). In order to be sure that all of the e-liquids had the same basic composition and varied only in terms of flavor components, we selected a single brand panel of e-liquids. Specifically, we tested first a sample with no additional flavor called “Base” and composed by a 50:50 ratio of pharma grade propylene glycol and vegetable glycerin. The other five samples contained a variable percentage (4–6%) of concentrated flavored blends. These flavors were chosen in order to represent the three main diffused categories: tobacco, mint and food flavors (Table 1).

To verify whether or not nicotine induces cell toxicity, each e-liquid was tested with (i.e., 18 mg/mL) and without nicotine.

### 2.2. E-CIG Condensed Aerosol (CAs)

**Figure 1:**
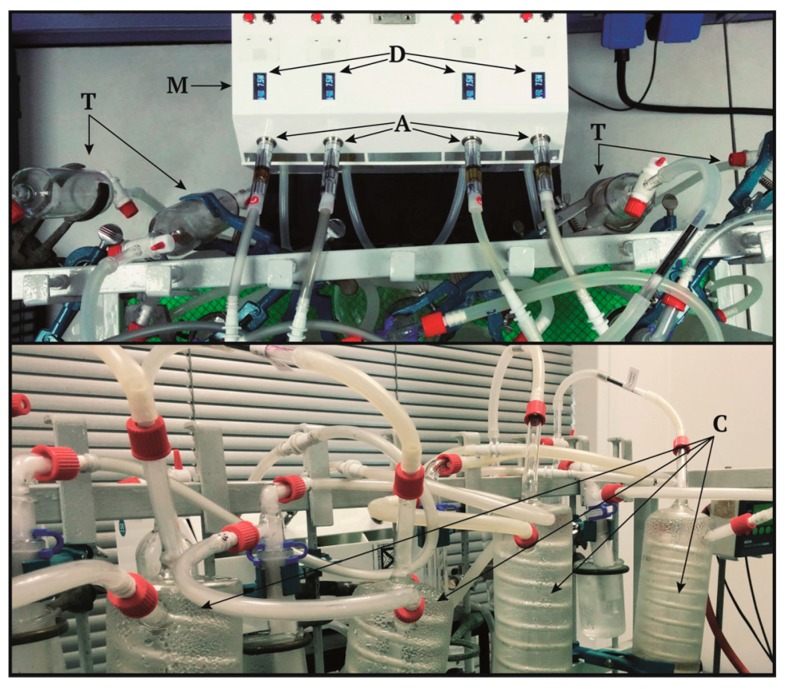
The Vaping machine, custom-made apparatus for generating and condensing vapors from e-CIGs. In the picture it is showed the four channel vaping machine (**M**) set with four atomizers (**A**) through 510 socket adapter. Each atomizer is controlled by one Evolv DNA 75 circuit (**D**) and is plugged to a collection system for toxicological studies (**T**) and a condensation cilinder (**C**) for chemical assays.

A prototype vaping machine equipped with an aerosol condensing trap was specifically designed and optimized for CAs testing (TRUSTiCERT Vaping Machine v2.0, TRUSTICERT SRL, Milan, Italy) (Figure 1). Details on the construction characteristics and operating conditions are reported in Supplementary Materials.

In order to simulate the stream contact with lung epithelial cells we set up an aspiration system connected to a flask containing 25 mL of cell culture medium (OPTIMEM 1% FBS). The flask was connected to a Kit iSimple Ribilio with C14 Passthrough 900 mah (SKU HWRB-162) that is one of the most sold e-CIG in Europe.

Each e-liquid has been tested in several atomizers with a fully charged battery. The weight of atomizer has been measured before and after vaporization procedure. Puff regime was set to 55 mL puff volume, 3 s draw, 60 s puff interval and 200 puff (estimated maximum of puffs per day 235, [26]).

The CAs obtained from the six samples (with and without nicotine) were immediately filtered using a Millipore filter (pores diameter 0.22 µm) to remove possible airborne bacterial contamination and stored at −20 °C until laboratory analyses.

### 2.3. Lung Cell Monocultures

The human lung adenocarcinoma cells A549 (ATCC^®^ CCL-185™) and NCI-H441 [H441] (ATCC^®^ HTB-174™) were maintained in OptiMEM1X (Gibco, Life Technologies, Monza, Italy) supplemented with 10% FBS (Gibco, Life Technologies, Monza, Italy) and 100 U/100 μg/mL of Pen/Strep (Euroclone, Milano, Italy) at 37 °C and 5% CO_2_. Human pulmonary microvascular endothelial cell line HPMEC-ST1.6R was received from Dr. Ronald E. Unger (Institute of Pathology, Medical University of Mainz, Johannes Gutenberg University, Mainz, Germany) and cultured in CellBIND Surface coated flasks (Corning, Corning, N.Y., USA ) in M199 medium (Euroclone, Milano, Italy) supplemented with 15% FBS (Gibco, Life Technologies, Monza, Italy), 2 mM Glutamax I (Sigma Aldrich, Milano, Italy), 25 μg/mL sodium heparin (Sigma Aldrich, Milano, Italy), 25 μg/mL endothelial cell growth supplement (Sigma Aldrich, Milano, Italy), and 100 U/100 μg/mL Pen/Strep (Euroclone, Milano, Italy) at 37 °C, 5% CO_2_.

### 2.4. In Vitro Model of the Alveolar-Blood Barrier (ABB): 3D Co-Culture of Epithelial and Endothelial Cells

An in vitro barrier consisting of a co-culture of epithelial and endothelial cells was set up as reported in [27]. In this model, two different compartments are defined: the apical one, composed by epithelial lung cells (NCI-H441), which are directly exposed to the e-CIGs condensed vapor, and the basal compartment composed of endothelial cells (HPMEC-ST1.6R) from the microcapillary pulmonary system.

Briefly, HPMEC-ST1.6R cells (9 × 10^4^ cells/cm^2^) were seeded on the lower surface of Transwell polyester membrane inserts (PET; 0.4 μm pore size, Costar; Corning, New York, NY, USA) coated with 0.2% gelatin (Sigma Aldrich) and placed in the incubator (37 °C, 5% CO_2_). After 2 h, the membrane inserts were turned upside down and placed in a 12-well plate with M199 complete endothelial medium. The day after, NCI-H441 (2 × 10^4^ cells/cm^2^) cells were seeded on the top surface of the Transwell inserts and cultured for 11–13 days until they have reached maximal transepithelial electrical resistance (TEER). To induce cells differentiation and tight junctions formation, essential characteristic for a functional barrier, NCI-H441 cells in co-cultures were treated with 1 μM Dexamethasone (Sigma Aldrich) starting from day 3 of co-culturing. The CAs (500 µL) were added to the apical compartment at day 12 and exposed for 24 h. At the end of the treatment supernatants in the apical and basal compartment were collected for ELISA analyses. TEER measurements were performed to investigate the barrier integrity and viability tests were assessed in order to investigate the cytoxicity of the tested compounds.

### 2.5. Cell Viability

Cytotoxicity assays were performed to screen the effects of the vapors on NCI-H441 and HPMEC-ST1.6R cells monocultures and co-cultures. MTT assay and Alamar Blue tests (alamarBlue^®^ Cell viability Reagent, Catalog nos. DAL1025) were performed according to manufacturers’ instructions. Cells viability MTT assay [3-(4,5-dimethylthiazol-2-yl)-2,5-diphenyltetrazolium bromide] (Sigma Aldrich, Milano, Italy) was used to measure cell viability in A549 cell line. After the treatment, supernatants were collected and stored at −80 °C, while cells were rinsed with PBS and MTT (final concentration 0.5 mg/mL) was added for 3 h. The medium was removed and MTT reduction product (formazan crystals) dissolved in 1 mL DMSO. The absorbance of each sample was assessed by Multiskan Ascent (Thermo Scientific Inc. Waltham, MA, USA) at 570 nm and it is proportional to cellular viability.

The Alamar Blue Reagent (Life Technologies, Monza, Italy) was used to assess the viability of the co-cultures. Alamar Blue is a water-soluble, non-toxic dye that yields a fluorescent signal and a colorimetric change when incubated with metabolically active cells. The oxidized form resazurin (blue) is reduced to a pink dye in the medium by cell activity.

In both MTT and Alamar Blue test the relative viability [%] related to the control samples (untreated cells) was calculated by:

Cell viability = (OD_sample_/OD_control_) × 100 (1)

The experiments were replicated at least three times and the results were expressed as mean percent ±SEM of viable cells in comparison to the controls (untreated cells).

### 2.6. Transepithelial Electrical Resistance (TEER) Measurements

The ABB integrity after the exposure to the CAs was determined by measuring the transepithelial electrical resistance (TEER) through an EVOM Volt Ohm Meter (World Precision Instruments, Berlin, Germany) equipped with an EndOhm 12 Chamber (World Precision Instruments, Berlin, Germany). TEER of polyester Transwell inserts coated with gelatine 0.2% (Sigma Aldrich), without cells, was measured and set as blank and then TEER was measured before (day 12) and after 24 h of exposure to CAs (day 13). TEER was measured as Ω × cm^2^ and calculated as follows:

TEER= (R_M_ − R_Blank_) × S (2)

where R_M_ is the experimental value of cell co-culture resistance, R_Blank_ the experimental value of the blank control and S the surface area of the filter membrane (1.12 cm^2^). Values of TEER were expressed as % ratio between TEER day 13/TEER day 12. The mean ±SEM of at least three independent experiments was presented.

### 2.7. Cytokines Release: IL-8 and MCP-1

The potential inflammatory effect induced by the exposure to CAs was evaluated by ELISA test. Cells supernatants from A549 monocultures and from apical and basal compartments of the co-cultures were collected after treatment and stored at −80 °C until the test was performed. The release of the pro-inflammatory mediator interleukin-8 (IL-8) and of the monocytes chemoattractant protein-1 (MCP-1) was investigated following manufacturers’ instructions (Novex, Life Technologies, Monza, Italy).

### 2.8. Statistical Analysis

Data are presented as mean and standard error of mean (SEM) of at least three independent experiments. Statistical analyses were performed using Sigma Stat 3.2 software, using unpaired *t*-test. Statistical differences were considered to be significant at the 95% level (*p* < 0.05).

## 3. Results and Discussion

### 3.1. Comparative Effects of CAs on Cells Monoculture

**Figure 2:**
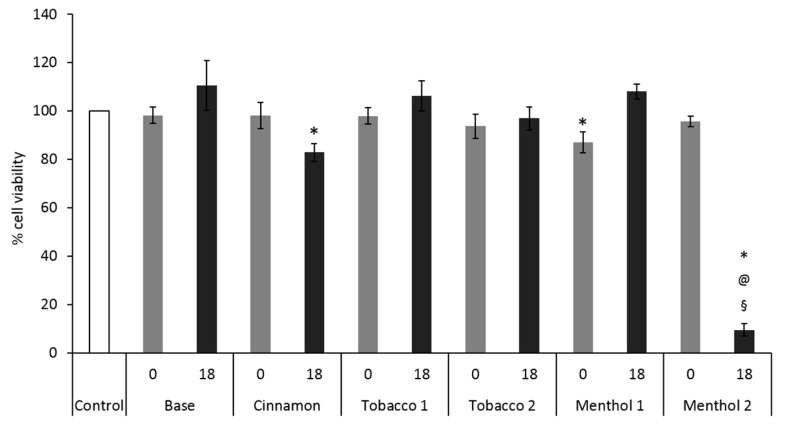
Cytotoxic effects of condensed aerosols (CAs) from e-liquid samples on monoculture A549 cells. MTT viability test was performed after 24 h of exposure to CAs belonging to e-liquids with (18 mg/mL, **black bars**) and without (**grey bars**) nicotine. Bars represent the percentage of viable cells with respect to the control (unexposed cells, white bar), considered as 100%. Data are presented as mean±SEM of at least 5 independent experiments. * *p* < 0.05; unpaired *t*-test over the control; @ *p* < 0.05; unpaired *t*-test over Base 0; § *p* < 0.05; unpaired *t*-test over Base 18.

**Figure 3:**
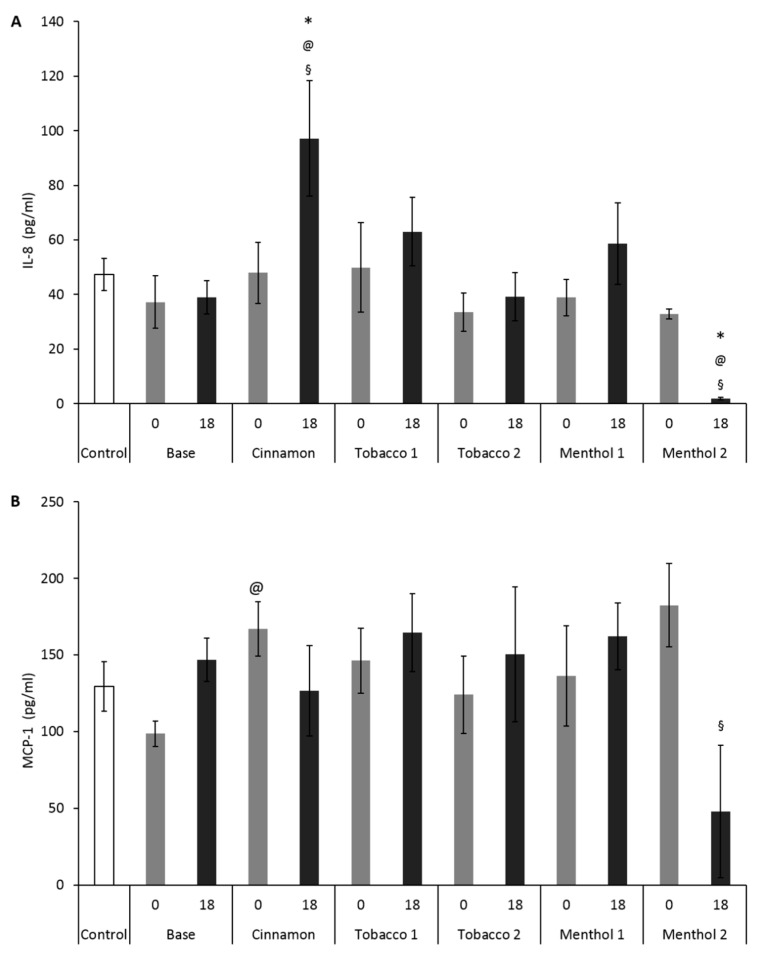
Pro-inflammatory cytokines released by monoculture A549 cells exposed to CAs from e-liquid samples with (18 mg/mL, **black bars**) and without (**grey bars**) nicotine; white bars represent control samples. (**A**), IL-8 release; (**B**), MCP-1 release. Bars represent the concentration (pg/mL) of the released protein. Data are presented as mean ±SEM of at least five different independent experiments. * *p* < 0.05; unpaired *t*-test over the control; @ *p* < 0.05; unpaired *t*-test over Base 0; § *p* < 0.05; unpaired *t*-test over Base 18.

The results obtained by testing CAs belonging to six e-liquid samples with and without nicotine on the monoculture A549 cells, suggest that they induce different cellular responses. In the case of Base, the e-liquid sample without flavor, no cytotoxic and pro-inflammatory effects were seen at both no and 18 mg/mL nicotine content (Figure 2 and Figure 3). Similar results were obtained for Tobacco 1 and Tobacco 2. This suggests that their CAs do not interact with cell viability and that no contribution to the toxicity was furnished by humectants and nicotine.

Conversely, slight to strong decrease in cell viability was observed after exposure to samples Cinnamon and Menthol 2 containing nicotine (Figure 2). In particular, the exposure to Menthol 2 induced cell death in the 90% of the cells. In absence of nicotine, these two flavors did not induce cytotoxic effects, suggesting that a synergistic action between flavor molecules and nicotine may take place. The synergistic effect of nicotine and flavors in e-CIG vapors is actually an open field of research and no literature is yet available. It is anyway known that the menthol contained in the mainstream smoke from conventional cigarettes may enhance the cellular uptake of PAHs and other tobacco-related carcinogens, finally producing a synergistic effect in altering metabolism [28] and promoting lung cancer [29]. These studies have been done on mentholated conventional cigarettes, where the tobacco component is determinant for the biological responses. Although no tobacco products are of course present in mentholated e-CIG, the capability of menthol to increase the bioavailability and cytotoxicity of nicotine and/or other molecules, deriving from the heating of the e-liquids, should not be excluded.

Since there are many concerns over the potential of e-CIGs to promote pulmonary inflammation, the capability of CAs to induce an inflammatory response was investigated. In the lung tissues of patients affected by chronic obstructive pulmonary disease (COPD), neutrophil infiltration is increased, as well as IL-8 (CXCL8, a key neutrophil chemoattractant) levels, and this may positively correlate with disease severity [30]. Furthermore, lung epithelial cells were seen to respond to several external stimuli by secreting specific chemo-attractants and pro-inflammatory cytokines, like MCP-1, in addition to IL-8, to activate the secondary response, consisting in neutrophils and macrophage recruitment. Finally, IL-8 is involved in endothelial dysfunction and MCP-1 contributes to the recruitment of monocytes to the endothelium, which may contribute in the development of vascular atherosclerotic processes [31]. For all these reasons, in this study we measured the upstream pro-inflammatory cytokines, IL-8 and MCP-1.

Our data showed that only the Cinnamon CA was able to induce a slight increase in the release of the pro-inflammatory cytokines IL-8 and MCP-1 in A549 cells. Specifically, Cinnamon with nicotine (Figure 3A) induced an increased secretion of IL-8 in A549 with respect to control cells and cells treated with CAs of Base with nicotine e-liquid. This suggests that the flavor occurring in Cinnamon could be responsible of the inflammatory response. The cinnamon flavor in e-liquids was previously reported to be cytotoxic [8], and it has been here confirmed that also its derived CAs are able to significantly decrease cell viability, while increasing inflammatory mediators release.

The strong decrease of IL-8 and MCP-1 release after exposure to Menthol 2 with nicotine was likely the consequence of the very high cell mortality registered by MTT. This result support the hypothesis that in alveolar epithelial cells, Cinnamon flavor is able to induce significant cytotoxic and pro-inflammatory response. Menthol 2, consisting in a combination of menthol with other flavoring molecules, such as carvone, (Table 1), displayed strong primary cytotoxic action (Figure 2A), which mined the capability of the cells to secrete inflammatory signals (Figure 3).

Leigh and co-workers [9] obtained similar results testing e-CIG CAs on NCI-H292 lung cells exposed at the air-liquid interface (ALI). In that case, strawberry flavor caused the highest cell mortality and release of inflammatory mediators. Similarly, our results, showed that CAs of Menthol 2 flavor cause significant reduction in cell metabolic activity and viability, without increasing the levels of cytokines release.

Our results are in line with the findings of Putzhammer et al. [16], who showed that herbal flavors contain a higher number of toxic compounds, which are likely responsible of the enhanced cytotoxicity. To verify this assumption, we used our experimental system to obtain and test CAs from e-liquids derived from plant extracts. The results highlighted the extremely high cytotoxic and pro-inflammatory effects of these compounds on A549 cells (see Supplementary Materials, Figures S1A and S2).

### 3.2. Comparative Effects of CAs on the ABB Model

The ABBs were used to test the CAs from samples containing nicotine, since they induced the most evident cytotoxic and pro-inflammatory effects on cell monocultures. CAs were administered when the TEER of the system was maximized. This was at 12 days of culture, when the TEER values reached the maximums of about 500–700 Ohm/cm^2^. At this time, cell junctions were differentiated and the culture represented a functional epithelial barrier [27].

**Figure 4:**
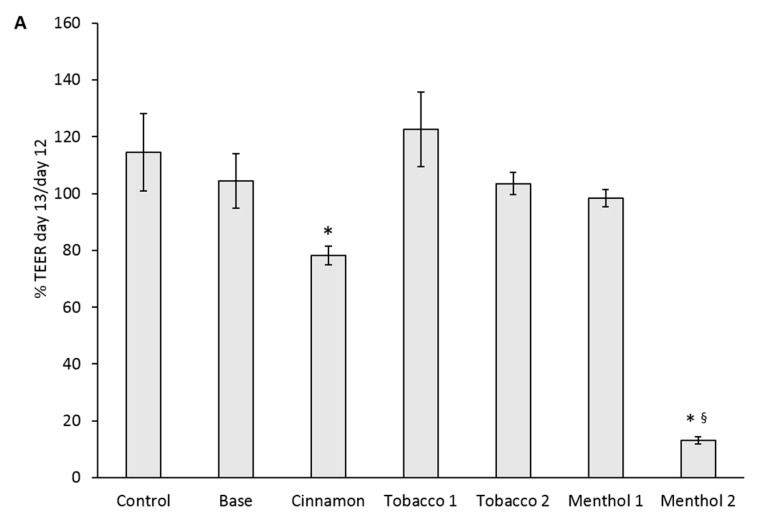

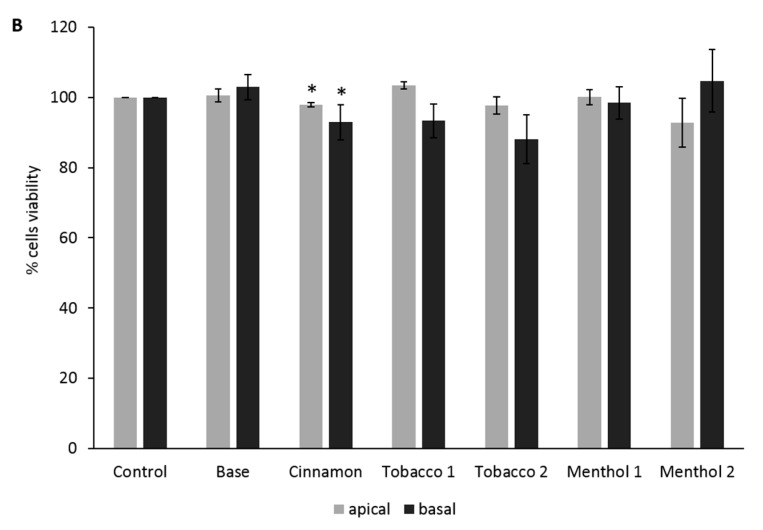
Barrier integrity and cell viability in the in vitro alveolar-blood barrier (ABB) model exposed to CAs from different e-liquids with 18 mg/mL nicotine. (**A**) trans-epithelial electrical resistance (% TEER day13/day12) measured across the barrier; (**B**) cells viability percentage measured in the alveolar NCI-H441 cells (apical, **grey histograms**) and in the endothelial HPMEC cells (basal, **black histograms**). Data are presented as mean ± SEM of at least three different independent experiments. * *p* < 0.05; unpaired *t*-test over the control (untreated cells); § *p* < 0.05; unpaired *t*-test over Base 18.

Results suggested that after 24 h of exposure of the 3D ABBs, Cinnamon and Menthol 2 significantly affected the barrier integrity, drastically reducing the TEER. No significant effects on TEER were observed after exposure to the other tested e-liquid CAs samples (Figure 4a).

Also, cell viability at both alveolar (apical) and endothelial (basal) cells significantly decreased after exposure to Cinnamon, while it did not occur for the other flavors, including Menthol 2 (Figure 4b). Since after Menthol 2 CA exposure cell viability did not result in being affected in ABB, differently to what observed in A549 monoculture, we can speculate that the co-culture system is less sensitive to the cytotoxic insult than the monoculture one. It has indeed been reported in literature that sub-confluent cell monocultures are more sensitive to xenobiotic exposure in respect to 3D co-culture conditions in which a functional ABB have been differentiated [32,33]. Nevertheless, the Menthol 2-induced TEER extreme reduction pointed out a strong effect on the ABB, likely consisting in the loss of cell junction integrity, but without significantly affecting cell metabolism at this stage. In acute exposure conditions, such an effect may be the consequence of a modulation of the tight junctions expression and/or cytoskeletal actin rearrangement, which may finally determine enhanced epithelial permeability. These aspects are of course worthy of further experimental evidences to be proved. Similar results have also been reported by Neilson and colleagues, who observed a loss in TEER induced by NJOY Menthol e-CIGs vapors in the EpiAirway™ system that did not correspond to a reduction in cell viability [15].

Based on our results, we can argue that Cinnamon and Menthol 2 may have different mode of action on the ABB. Moreover, the positive response obtained from the TEER measurement, even in absence of cell viability reduction, suggests the effectiveness of this marker in discriminating the precocious effects achievable from CAs exposure.

**Figure 5:**
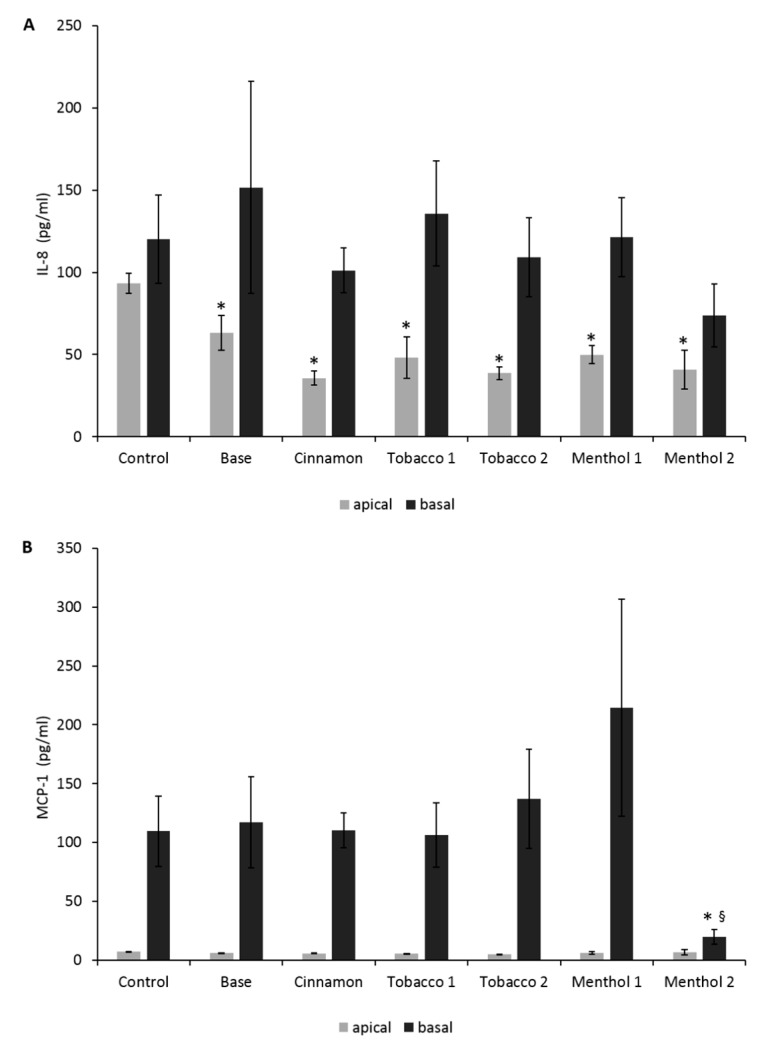
Pro-inflammatory cytokines release in the ABB model. (**A**) IL-8 release by the alveolar NCI-H441 cells (apical, grey bars) and by the endothelial lung microvascular endothelial cells (HPMEC) cells (basal, black bars); (**B**) MCP-1 release by alveolar NCI-H441 cells (apical, grey bars) and by the endothelial HPMEC cells (basal, black bars). Bars = pg/mL of IL-8 and MCP-1 released by co-cultures. Data are presented as mean ± SEM of at least three different independent experiments. * *p* < 0.05; unpaired *t*-test over the control; § *p* < 0.05; unpaired *t*-test over basal Base 18.

No observable increase in IL-8 and MCP-1 secretion was recorded in the ABBs exposed to all of the CAs (Figure 5a,b). The only appreciable effect was the significant decrease in IL-8 release in the apical side of the transwell system, suggesting no activation of pro-inflammatory pathway by CAs at the ABB level and a possible anti-inflammatory action of nicotine It was independent from the flavor contained, since also Base sample (Table 1), containing nicotine, was able to induce a comparable effect (Figure 5a).

Nicotine has both pro- and anti-inflammatory effects depending on the in vitro model used [34], and it induces cell proliferation and angiogenesis [35], suggesting that it can be responsible for the decreased inflammatory response in cells exposed to different CAs.

When considering the CAs from natural plant extracts, only Sambuca flavor induced severe results in the ABB model. All of the toxicity markers studied—TEER, cell viability of epithelial and endothelial cells, cytokines release by epithelial, and endothelial cells—revealed strong positive results after exposure to Sambuca aerosols (see Supplementary Materials, Figures S3A,C and S4A).

Similarly, the condensed smoke produced by conventional cigarettes was able to drastically alter all of the toxicity endpoints considered (see Supplementary Materials, Figures S1B, S3B,D and S4B).

These observations support that the experimental approach adopted in the present study is reliable in efficiently discriminating the contribution of different e-liquid flavorings to the in vitro inhalation toxicity.

## 4. Conclusions

In a recent paper, Hiemstra and Bals [36] reviewed the state of the art of the in vitro and in vivo approaches used for the e-CIG safety assessment. They concluded that the studies demonstrated the less toxicity of the e-CIG with respect to the conventional tobacco products, but the effects were not due to the nicotine content only. They also underlined the lack of standardized toxicological tests in this field. By using two different in vitro models for the e-CIG inhalation toxicity screening, we were able to confirm that aerosols from e-CIG are less hazardous than condensed smoke from conventional cigarettes, but some flavorings significantly contribute to induce cytotoxic effects in lung cells. In particular, the flavorings based on natural plant extracts (e.g., Sambuca), the menthol-based and the food-related ones (e.g., Cinnamon) significantly affected the lung cell viability and the respiratory barrier integrity. The A549 monoculture and the 3D-ABB model, made of alveolar epithelial and microvascular endothelial cells, were able to discriminate the effects of the different flavorings under the experimental conditions adopted in this work. The easy and cost-effective assays for cell viability in the monoculture and TEER measurement in the ABB model resulted to be predictive for the toxicological pre-screening of the vapors deriving from several e-liquids. This condition underlines the possibility to include both simple and more complex 3D in vitro systems in the framework of the e-CIG toxicological safety assessment.

3D models of the epithelial barriers and organoids are at the forefront of the toxicological studies and in the very next future they will likely help in the further reduction of the animal experimentation and in a more detailed comprehension of the mechanistic aspects of the organ toxicity. Their use in the field of e-CIG experimentation would represent a great benefit for both the scientific community and regulatory agencies.

The refinement and harmonization of the vaping machines and conditions, as well as the identification of in vitro standardized models and assays are urgently required to match the necessity of the companies and the regulatory bodies, which basically consists in low-cost, time saving and reproducible methods.

In addition to the identification of reference toxicological models and methods, it is anyway mandatory to further explore the mechanisms of action of the huge variety of products already in commerce. It should be also considered that the consumer has the possibility to generate new unknown blends by mixing the commercial references, as well as the potential health hazard deriving from the prolonged exposure and abuse of e-CIGs.

## Figures and Tables

**Table 1:** Composition of the tested e-liquids. For each sample, both no and 18mg/mL nicotine were analysed. PG: pharma grade propylene glycol, VG: vegetable glycerin.

Identifier	Nicotine (mg/mL)	E-Liquid Composition (PG:VG:Water:Blend)	Blend Composition
Base	0	50:40:10:0	N.A.
	18		
Cinnamon	0	50:40:7:3	Propylene glycol (57-55-6) 80% Water (7732-18-5) 18% Cinnamaldehyde (104-55-2) 1.5% Cinnamon oil (84961-46-6) 0.5%
	18		
Tobacco 1	0	50:40:7:3	Propylene glycol (57-55-6) 90% Water (7732-18-5) < 5% Dimethylhydroxy Furanone (3658-77-3) < 3% Triacetin (102-76-1) < 3% Cocoa Extract (84649-99-0) < 1% Hepta-2,4-dienal trans trans (4313-03-5) < 1% Racementhol (89-78-1) < 1% others < 0.1%
	18		
Tobacco 2	0	50:40:7:3	Propylene glycol (57-55-6) 90% Water (7732-18-5) < 5% Triacetin (102-76-1) < 3% Methylcycopentenolone (80-71-7) <1% Vanillin (121-33-5) < 1% Ethyl Lactate (97-64-3) < 1% Ethyl Vanillin (121-32-4) < 1% others < 0.1%
	18		
Menthol 1	0	50:40:3:7	Propylene glycol (57-55-6) 90% Menthol (89-78-1) 10%
	18		
Menthol 2	0	50:40:4:6	Propylene glycol (57-55-6) 90% Menthol (89-78-1) 5% Carvone (99-49-0) 5%
	18		

## Supplementary Materials

The following are available online at www.mdpi.com/1660-4601/14/10/1254/s1, Figure S1: Cytotoxic effects of CAs from natural plant extracts e-liquids and CSE on A549 cells, Figure S2: Pro-inflammatory cytokines release by A549 cells exposed to CAs from natural plant extracts e-liquids, Figure S3: Barrier integrity and cell viability in the in vitro ABB model exposed to CAs from natural plant extracts e-liquids and CSE, Figure S4: Interleukin-8 release in the ABB model.
