# Supplementary material for: Lung Toxicity of Condensed Aerosol from E-CIG Liquids: Influence of the Flavor and the In Vitro Model Used
Source: Int J Environ Res Public Health. 2017 Oct 20;14(10):1254. doi: 10.3390/ijerph14101254 (PMC5664755; doi:10.3390/ijerph14101254)
Supplement: Supplementary file 1 [file ijerph-14-01254-s001.pdf]

Inhalation toxicity of condensed aerosol from e-CIG liquids: influence of the flavor and the *in vitro* model used.

Rossella Bengalli[1], Emanuele Ferri[2], Massimo Labra[3], Paride Mantecca.[1*]

[1] *University of Milano Bicocca, Department of Earth and Environmental Sciences, Research Center POLARIS (Particulate Matter and Health Risk), 1 piazza della Scienza, 20126 – Milan, Italy*

[2] *TRUSTICERT SRL, via Mazzini 18/C, 22036 - Erba (CO), Italy*

[3] *University of Milano Bicocca, Department of Biotechnology and Biosciences, 2 piazza della Scienza, 20126 – Milan, Italy*

* *Correspondence: paride.mantecca@unimib.it; Tel.: +39-02-64482916*

Supplementary Material

Materials and methods

1. Description of the TRUSTiCERT Vaping Machine v2.0

A Vaping Machine was custom built to independently test 4 e-cigarette atomizers by simulating the vape thanks to Arduino Uno and a dedicated software. Each testing position is compostED by an Evolv DNA75 circuit, a 18650 battery and a solenoid electrovalve. The DNA75 allows to manually set the wattage, control the temperature of the coil and read the resistance of the coil. Every kind of 18650 battery can be used to power the DNA75 (for our tests we used NiteCore 18650D 3100 mah). The DNA75 firing signal is remotely controlled by an Arduino Uno that adjust the time and frequency of fire. The Vaping Machine is compatible with 510 atomizers and 510 adapters. Adapters are installed with a 45° inclination angle in order to optimize vaping topology that simulates the real position and to minimize the possibilities of dry puffs. The electrovalve, connected to a vacuum pump, simulates the human inhalation: the valve is normally closed, when the valve receives a signal, it opens and the pump can draw the vapour.

The opening signal is controlled by Arduino Uno and it is synchronized with the firing signal to perfectly simulate the vaping. The Arduino Uno software can be configured by a Web App that allows setting 3 parameters of the experiment: number of firing, firing time and interval time between firing.

The four separate channels are asynchronous, in order to not divide the flow of the air maintaining the correct pressure between the channels during the test.

Svapo Configurator is used to set up firing time, number of firing and the interval between firing. Each EvolvDNA is set up using the related +/- buttons to 35 Watt which correspond to optimal setting for the combined electronic cigarette used in combination with e-liquids to be tested.

The hardware to test is filled with the proper e-liquid, following the instruction of the producer, then plugged to the connector of the Vaping Machine. Each atomizer is connected to a trap that collects the

emissions and the output of the trap is connected to the related inlet pipe of the Machine. Each trap is prefilled with 25 ml of culture medium. The vacuum pump is turned on and the manometer is set to 800 mBar.

At the end of the protocol each atomizer is checked for burn signs on the wick material around the coil. If burned spots are found the medium is discarded and the protocol is repeated from the beginning with a new atomizer.

2. Cigarette smoke extract (CSE) preparation and CAs from natural plant extracts

Cigarette smoke extract (CSE) samples deriving from 1 (CSE 1) or 5 (CSE 5) conventional cigarettes were collected from conventional cigarette (we selected the brand most sold in Italy with nicotine 0.6 mg, tar 8 mg and CO 9 mg). For such experiment, a second machine has been equipped with conventional cigarettes directly plugged to the collection tubes. The puff regime was the same for EC. Twelve puffs were enough for completely burning a new cigarette.

Two refill fluids deriving from natural plant extracts were purchased from the market. The samples were indicated as Sambuca and Tangerine and the content of nicotine in each refill fluid is 8,55 mg/ml.

CAs were collected and prepared following the same procedure and puff regime described in the paragraph 'Materials and methods, 2.2 E-CIG condensed aerosol'.

Results

<u>Supplementary Figure S1</u>

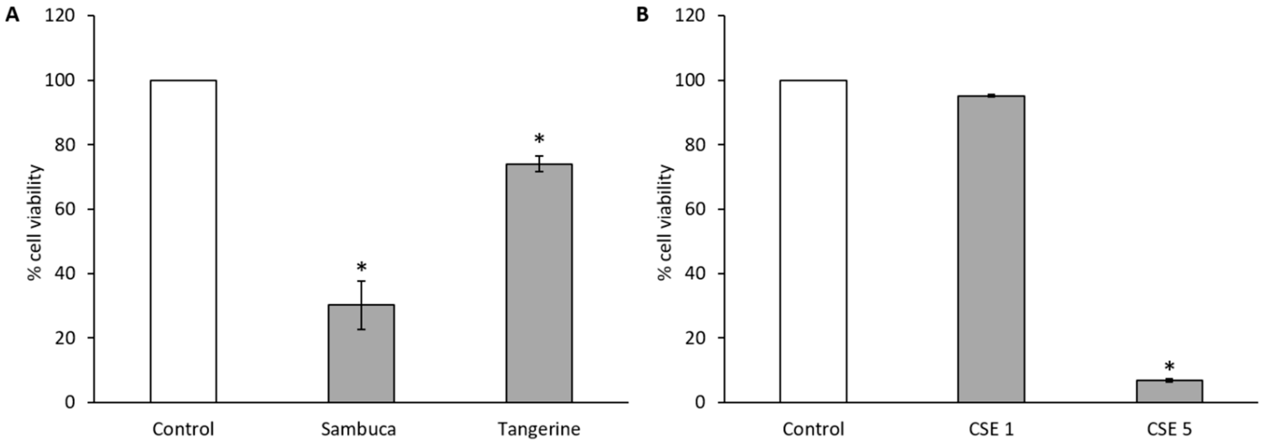

*Fig. S1. Cytotoxic effects of CAs from natural plant extracts e-liquids and CSE on A549 cells. MTT viability test was performed after 24h of exposure to CAs from Sambuca and Tangerine e-liquids (A) or from CSE of 1 or 5 conventional cigarettes (B). Histograms represent the percentage of viable cells in respect to the control (unexposed cells, white bar), considered as 100%. Data are presented as mean±SEM of at least 3 independent experiments.*p<0,05; unpaired t-test over the control.*

Supplementary Figure S2

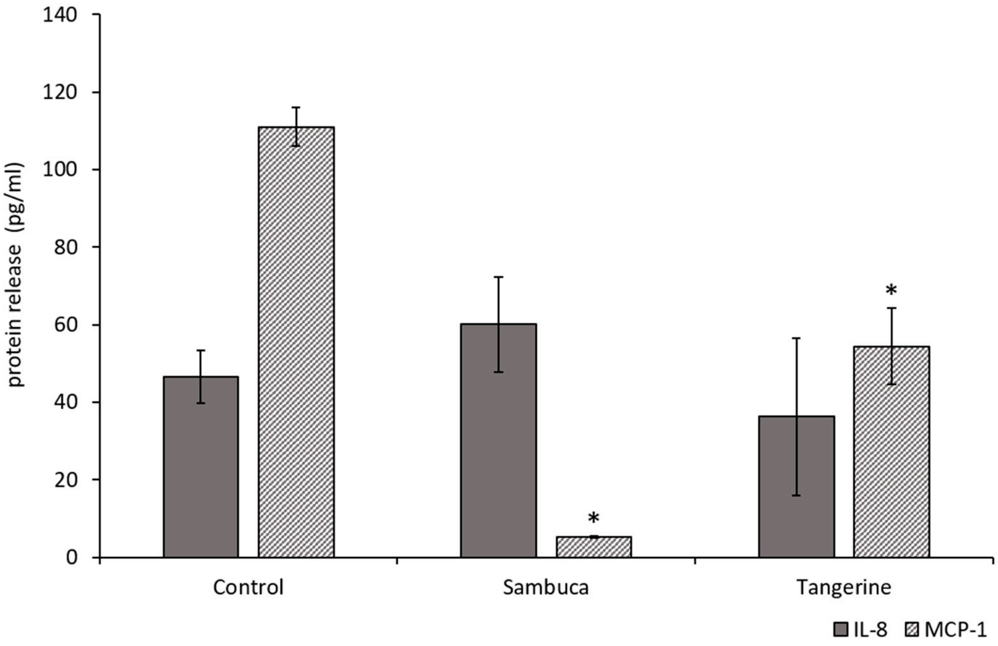

*Fig. S2. Pro-inflammatory cytokines release by A549 cells exposed to CAs from natural plant extracts e-liquids. Histograms represent the concentration (pg/ml) of the released protein. Grey bars represent IL-8 release; dashed bars indicate MCP-1 release. Data are presented as mean±SEM of at least 3 different independent experiments.*p<0,05; unpaired t-test over the control.*

Supplementary Figure S3

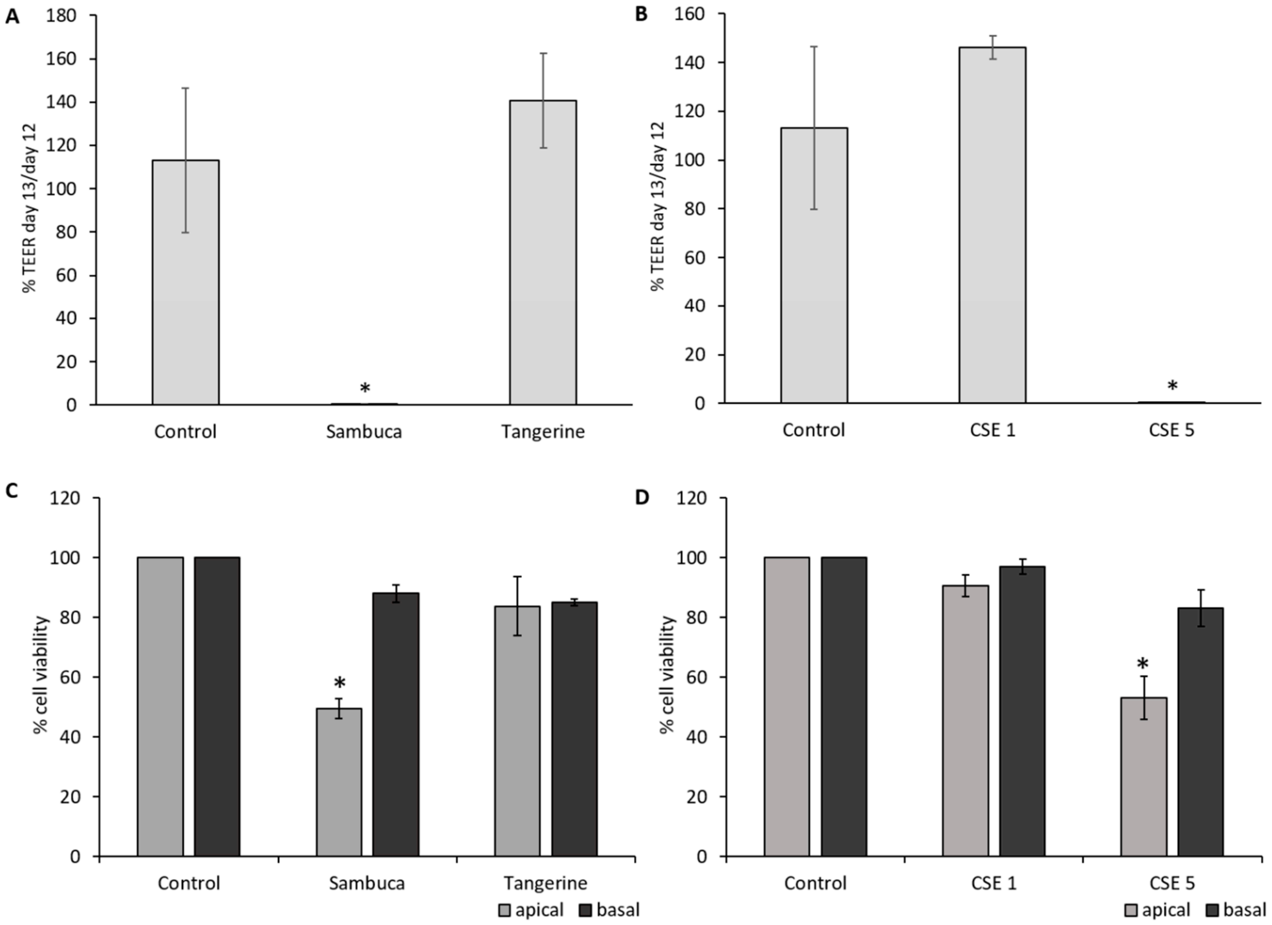

*Fig. S3. Barrier integrity and cell viability in the in vitro ABB model exposed to CAs from natural plant extracts e-liquids and CSE. A and B, trans-epithelial electrical resistance (TEER, Ohm/cm^2) measured across the barrier; C and D, cell viability percentage measured in the alveolar NCI-H441 cells (apical, grey histograms) and in the endothelial HPMEC cells (basal, black histograms). Data are presented as mean±SEM of at least 3 different independent experiments.*p<0,05; unpaired t-test over the control (untreated cells).*

<u>*Supplementary Figure S4*</u>

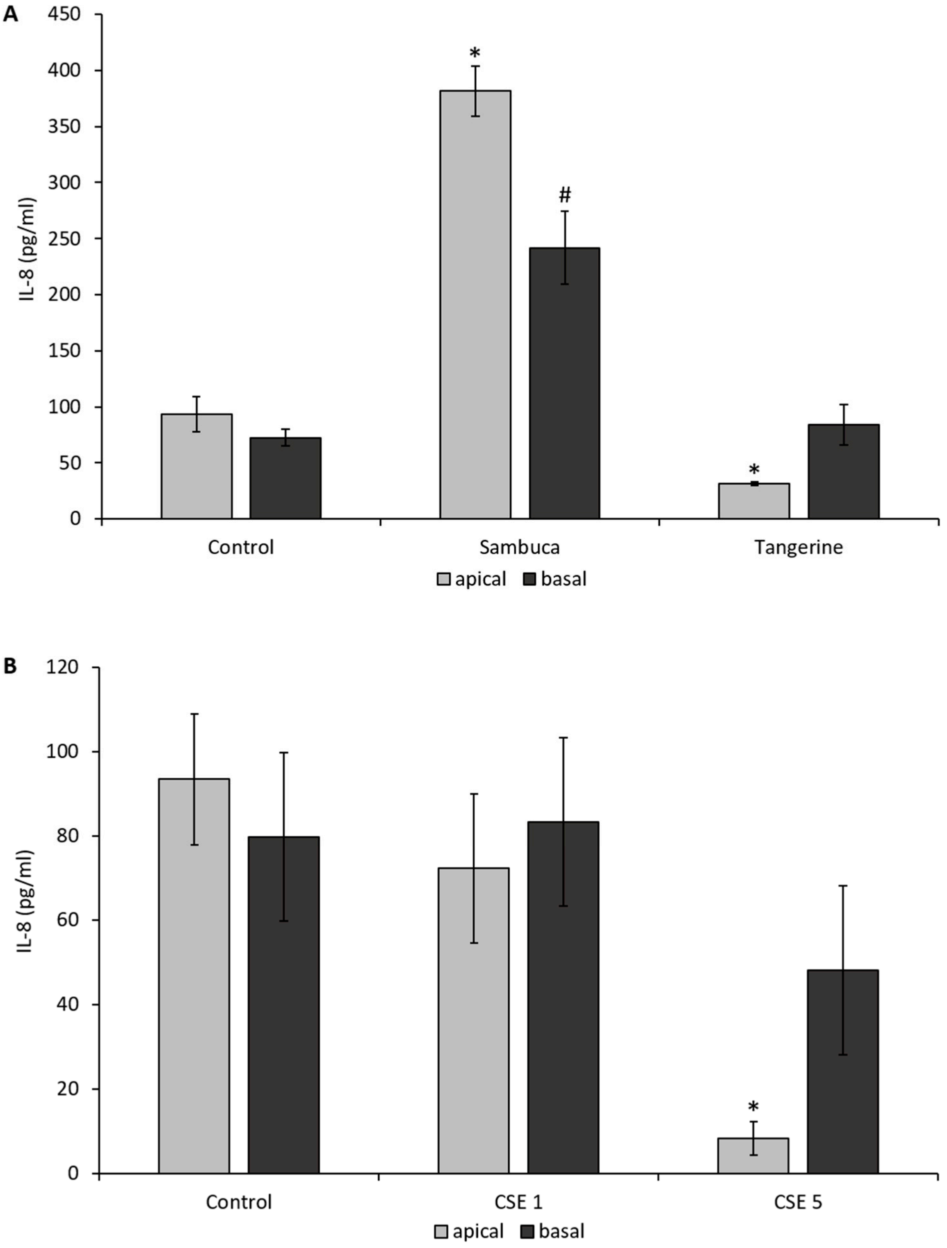

Fig. S4. Interleukin-8 release in the ABB mode). A, IL-8 release by the alveolar NCI-H441 cells (apical, grey bars) and by the endothelial HPMEC cells (basal, black bars) exposed to CAs from natural plant extracts e-liquids (Sambuca and Tangerine); B, IL-8 release by alveolar NCI-H441 cells (apical, grey bars) and by the endothelial HPMEC cells (basal, black bars exposed to CSE from 1 (CSE 1) and 5 conventional cigarettes (CSE 5). Bars= pg/ml of IL-8 and MCP-1 released by co-cultures.
